# A Scoping Review of the Impact of COVID-19 on Kidney Transplant Patients in the United States

**DOI:** 10.7759/cureus.35725

**Published:** 2023-03-03

**Authors:** Monica Karas, Isabel Bernal, Oscar Diaz, Ola Alshammari, David Baggett, Thomas Bronk, Siam Chawdhury, Adi Eylon, Evelyn Garcia, Kyiana Haughton, Breanne Kothe, Andrew M Joseph, Robin J Jacobs

**Affiliations:** 1 Osteopathic Medicine, Dr. Kiran C. Patel College of Osteopathic Medicine, Nova Southeastern University, Fort Lauderdale, USA; 2 Osteopathic Medicine, Dr. Kiran C. Patel College of Osteopathic Medicine, Nova Southeastern University, Davie, USA

**Keywords:** risk factors, sars-cov-2, coronavirus, treatment protocols, waitlist, nephrology, transplantation, kidney transplant, covid-19

## Abstract

SARS-CoV-2, responsible for the COVID-19 pandemic, is a highly infectious virus that quickly became and continues to be a public health emergency, given the severe international implications. Immunocompromised patients, such as those undergoing kidney transplantation, are at an increased risk for severe illness from COVID-19 and require hospitalization for more aggressive treatment to ensure survival. COVID-19 has been infecting kidney transplant recipients (KTRs), affecting their treatment protocols, and threatening their survival. The objective of this scoping review was to summarize the published literature regarding the impact of COVID-19 on KTRs in the United States in terms of prevention, various treatment protocols, COVID-19 vaccination, and risk factors. The databases such as PubMed, MEDLINE/Ebsco, and Embase were used to search for peer-reviewed literature. The search was restricted to articles that were published on KTRs in the United States from January 1, 2019, to March 2022. The initial search yielded 1,023 articles after removing duplicates, leading to a final selection of 16 articles after screening with inclusion and exclusion criteria. Four domains emerged from the review: (1) impacts of COVID-19 on performing kidney transplants, (2) impacts of COVID-19 vaccinations on KTRs, (3) outcomes of treatment regiments for KTRs with COVID-19, and (4) risk factors associated with an increased mortality rate of COVID-19 in KTRs. Waitlisted patients for kidney transplants had a higher risk of mortality compared to nontransplant patients. COVID-19 vaccinations in KTRs are found to be safe, and the immune response can be improved by placing patients on a low dose of mycophenolate before vaccination. Withdrawal of immunosuppressants showed a mortality rate of 20% without increasing the rate of acute kidney injury (AKI). There is evidence to support that kidney transplantation with the accompanying immunosuppressant regimen can provide KTRs with better COVID-19 infection outcomes compared to waitlisted patients. Hospitalization, graft dysfunction, AKI, and respiratory failure were the most common risk factors that increased the risk of mortality in COVID-19-positive KTRs. Withdrawing KTRs from immunosuppressive drugs increased the mortality rate. Further studies are needed to investigate the effects of specific drugs and dosages on the severity and mortality rate of COVID-19 in KTRs.

## Introduction and background

SARS-CoV-2, responsible for the COVID-19 pandemic, is a highly infectious virus that quickly became and continues to be a public health emergency, given the severe international implications in the sectors of healthcare, economy, and quality of life. COVID-19 can manifest differently in each person, ranging from asymptomatic to severe illness [1]. Symptoms include fever, chills, cough, congestion, dyspnea, sore throat, fatigue, myalgia, loss of smell/taste, and headache [1]. More severe complications could result in organ failure and death [2]. While anyone can become infected, certain populations may be at an increased risk for severe disease [3]. Immunocompromised patients, such as those undergoing kidney transplantation, are among those at an increased risk for severe illness and may require hospitalization for aggressive treatment for survival [3]. Furthermore, studies have also shown that the humoral response to current COVID-19 vaccines has been poor in this population, leading to a greater risk of exposure [4,5]. COVID-19 has been infecting kidney transplant recipients (KTRs), affecting their treatment protocols, and threatening their survival. Therefore, the purpose of this scoping review was to summarize the published literature regarding the impact of COVID-19 on KTRs in the United States in terms of prevention, various treatment protocols, COVID-19 vaccination, and risk factors.

COVID-19

In September 2021, 221 million individuals were infected with COVID-19 worldwide [6]. The United States accounted for 39 million of the total cases reported, with a death toll of 654,000 [7]. In the United States, the first case of COVID-19 was reported on February 2020. By March 2020, all 50 states had reported at least one case of the virus [8]. The high transmission rate of COVID-19 is due to its elevated reproductive ratio of 2.2 to 2.5 [9]. Although highly infectious, COVID-19’s infectivity may not be prominent until seven to 10 days after the onset of symptoms [10]. However, isolation and quarantine measures can reduce the transmission rate of the virus [10].

The importance of prevention cannot be understated; COVID-19 can remain active in sneeze droplets for three hours when artificially aerosolized [11]. In two years since the pandemic, major pharmaceutical companies like Pfizer and Moderna have mass-produced vaccines for the public, which are mRNA-based vaccines that produce an immunological response to the S protein produced by COVID-19 [12]. Moderna’s clinical trials revealed that its vaccine efficacy is estimated to be 94.1% [12]. Remdesivir, an antiviral medication, appears to be, for the moment, the gold standard for therapy [13].

KTRs and immunosuppressive therapy

KTRs regularly take immunosuppressive drugs to ensure a successful response to allograft and prevent transplant rejection [14]. Over the last few decades, there has been a steady increase in kidney transplants in the United States due to the rise in end-stage renal disease (ESRD). This rise is, in part, due to the combination of the obesity epidemic and the increase in both diabetes and hypertension [14]. Due to the scarce availability of renal allograft, patient selection for kidney transplants is a robust and strict process. To be considered for renal transplant, a candidate must have either a glomerular filtration rate of less than 20 mL/minute or be receiving hemodialysis [14]. Other factors, such as age, comorbidities, and previous medical noncompliance are also weighed into the decision [14].

Recovery from kidney transplant surgery is an area that is not well documented. Although it is widely used in many surgical subspecialties, the enhanced recovery after surgery protocol (ERAS) has just recently been implemented into renal transplants [15]. ERAS, when utilized on patients that have undergone previous renal transplants, was found to decrease morbidity as well as future readmission rates [16]. Following ERAS on patients postoperatively could be considered the gold standard in future guideline alterations [16]. In patients who have to be readmitted, the underlying culprit appears to be urinary tract infections [16]. These infections often manifest after the transplant procedure due to the patient’s immunocompromised state [16].

Immunosuppressive therapy begins immediately after a successful surgical intervention. The goal is to inhibit three important aspects of T-cell activation and proliferation [17]. After the surgery, patients must remain on immunosuppressive therapy for the rest of their lives [17]. Without it, the risk of tissue rejection is inevitable [17]. While corticosteroids are widely used as an immunosuppressive therapy to reduce transplant rejection risk, the associated long-term side effects may prove to be detrimental or fatal to the patient [18]. Because of this, calcineurin inhibitors have become the mainstay pharmacological agents used in posttransplant therapy [18]. Their mechanism of action inhibits the transcription of IL-2, which is needed for T-cell growth [17]. New immunologic therapy and protocols are currently being studied to assess their efficacy with respect to that of calcineurin inhibitors; however, their long-term side effects are yet to be determined [18].

Immunosuppressive drugs that are used to manage KTRs can potentially cause lymphopenia, which is commonly found in patients with COVID-19 [19,20]. Additionally, there is a negative association between lymphocyte count and disease severity [19,20]. As such, KTRs are at a higher risk for severe COVID-19 infections due to their suppressed immune system’s inability to combat the infecting pathogen [19,20].

Previous research on KTRs and COVID-19 

Since KTRs are chronically immunosuppressed, this issue of COVID-19 prevention has become significant [21]. Although the COVID-19 vaccines have been shown to effectively prevent human-to-human transmission, many expedited studies examining the efficacy of the current vaccines largely excluded transplant recipients from their subject population [22]. Studies of the mRNA COVID-19 vaccine in the immunocompromised transplant recipient population have shown impaired immunological response to the vaccine. In one preliminary study, only 10.8% of vaccinated KTRs showed positive serologic responses 28 days after receiving their first injection of the Moderna mRNA vaccine [5]. Because of this, transplant specialists are urging vaccinated recipients to remain diligent with social distancing, hygiene, and masking precautions until they have had definitive serology testing proving sufficient immunologic response [4,22].

Regarding the impact of a COVID-19 infection on KTRs, laboratory results are similar between transplant and nontransplant recipients [21]. White blood cell and platelet counts remained within normal limits in both populations [21]. Despite this, the transplant population was found to have a higher incidence of relative neutropenia, along with an elevated level of both serum ferritin and erythrocyte sedimentation rate [21]. In addition, patients infected with COVID-19 who were seen in the intensive care units often had acute kidney injuries presenting as nephritis and proteinuria [21]. While the cause of this is still not fully understood, one such theory suggests that COVID-19 potentially targets the kidney at the angiotensin-converting enzyme 2 (ACE-2) receptors in both endothelial cells and podocytes [21]. Although the majority of these patients had their acute kidney injury (AKI) resolved, renal involvement was found to be associated with a higher incidence of mortality [21].

Studies have shown that COVID-19 vaccinations do not provide a sustainably positive serologic response in KTRs [4,22]. It is unknown if COVID-19 is immunomodulatory and can trigger acute allograft rejection [21]. Currently, there is a research gap regarding the treatment and prevention of COVID-19 in KTRs. Literature published in this area has urged for an increase in this research due to both a drastic spike in COVID-19 infection rates in KTRs and the recent discovery of kidneys being one of the main targets of infection [21]. New immunologic therapy and protocols are currently being studied to withdraw the use of Calcineurin inhibitors; however, the long-term effects have not been elucidated and could potentially be more harmful to the patient [18]. As such, the purpose of this scoping review was to summarize the published literature regarding the impact of COVID-19 on KTRs in the United States in terms of prevention, various treatment protocols, COVID-19 vaccination, and risk factors.

## Review

Materials and methods

This scoping review was conducted to gather and synthesize the published literature addressing the effects of COVID-19 on KTRs. Following the guidelines of the Preferred Reporting Items for Systematic Reviews and Meta-Analysis (PRISMA), peer-reviewed literature involving COVID-19, kidney transplants, prevention, and treatment protocols in KTRs was searched using databases. The databases such as PubMed, MEDLINE/Ebsco, and Embase were used to search for peer-reviewed literature. The search was restricted to articles that were published on KTRs in the United States from January 1, 2019, to March 2022. Boolean operators were used combining the following terms: “Kidney Transplant” AND “SARS-CoV-2” OR “Covid-19” OR “coronavirus” AND “Treatment” OR “Protocol.” Inclusion criteria included kidney transplants, hospitalized patients, the United States, and COVID-19. Exclusion criteria were other types of transplants, outside the United States, Middle East Respiratory Syndrome (MERS), other respiratory viruses, systemic reviews, scoping reviews, case studies outpatient, ESRD, chronic kidney disease, and multi-organ transplants.

**Figure 1:**
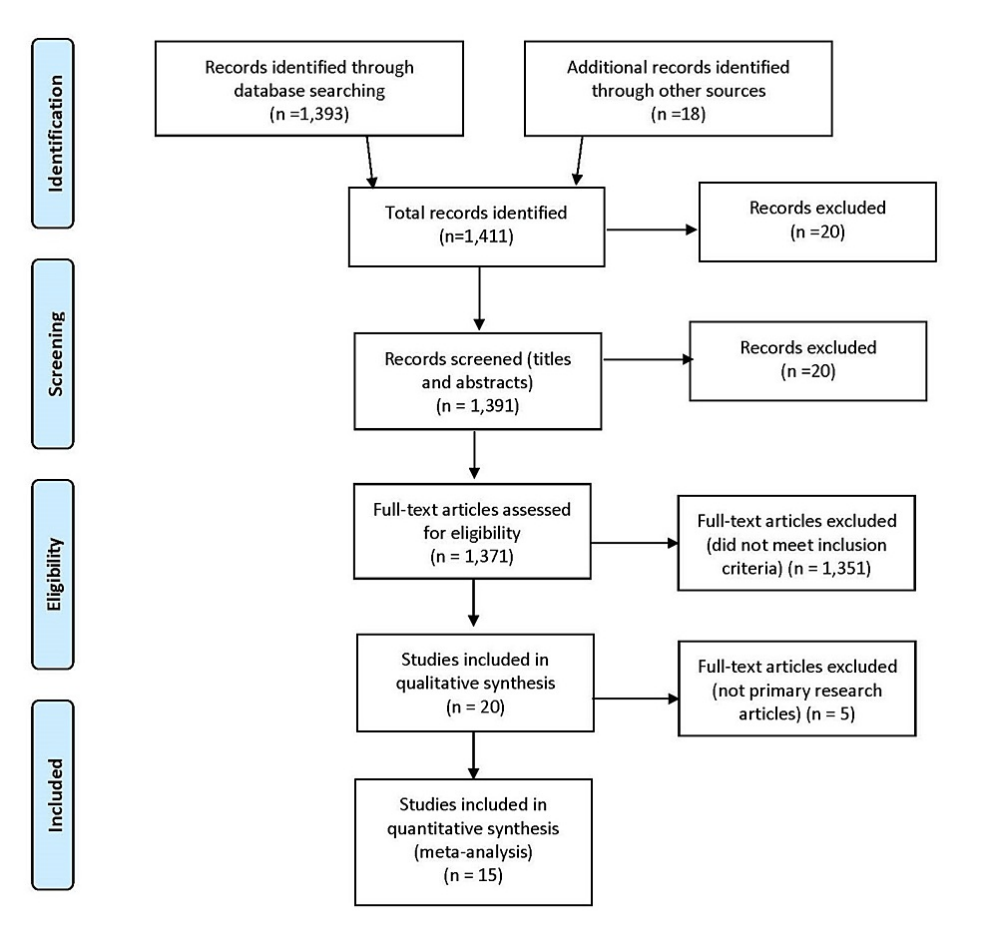
PRISMA flowchart describing the study selection pathway. PRISMA, Preferred Reporting Items for Systematic Reviews and Meta-Analysis

Using the PRISMA method for article selection and screening, the initial search yielded 1,037 articles after removing duplicates. Next, 1,021 articles were screened, and 20 articles were excluded due to lack of article access. An additional 990 articles were excluded due to a lack of full-text access. After screening based on the inclusion and exclusion criteria and critical appraisal of the reports, a total of 15 articles were selected for the final review (Figure 1). 

Results

Domain 1: Impact of COVID-19 on Performing Kidney Transplants

The overall impact of COVID-19 on performing kidney transplants has been evaluated in two studies in this review [23,24]. In one study where patients who were waitlisted for a kidney transplant, irrespective of the overlapping comorbidity burden shared with KTRs, fared worse than their counterparts who were organ recipients [23]. Waitlisted patients were at higher risk for both requiring hospitalization and mortality. Further analysis of the data demonstrated that waitlist status, age, and male sex were independent mortality risk factors, with requiring intubation to be an indicator of a poor prognosis for the patient. Another study followed a group of KTRs postoperatively and despite being at higher risk of infection from COVID-19, none of the patients contracted COVID-19 [24]. These studies demonstrated a benefit to transplantation as KTRs were less likely to require hospitalization and ultimately fared better than the patients who were awaiting a transplantation operation.

Domain 2: Impact of COVID-19 Vaccinations on KTRs

Two studies that assessed the efficacy of obtaining an antibody response from the mRNA COVID-19 vaccine on KTRs suggested that transplant recipients did not have a similar vaccine response to the general population [25,26]. Further, the type of immunosuppressive regimen that the KTRs were on might affect the vaccine response [25,26]. Vaccine response for adolescent KTRs performed slightly better than adult KTRs [25]. Another study noted similar effects of differences among patients' immunosuppressant regimens and the immunogenicity of the mRNA vaccinations in KTR [26]. The authors of that study found that only 25% of KTRs who were vaccinated against COVID-19 generated a detectable amount of antispike protein antibodies, and only two of 17 patients receiving mycophenolate as part of the immunosuppressant regimen developed a detectable amount of antispike protein antibodies [26]. That same study also found that KTR patients receiving Belatacept as part of their immunosuppressant regimen did not develop any detectable level of antispike protein [26]. Both studies also demonstrated the safety and efficacy of mRNA COVID-19 vaccines; they were well tolerated with no reported adverse effects [25,26]. It was also confirmed that response to COVID-19 vaccination in KTRs showed on average less effectiveness compared to the general population [25,26]. Both studies included antispike protein antibody levels; however, only one of the two studies included the antibody level before giving the vaccine to the participants.

Domain 3: Outcomes of Various Treatment Regimens for KTRs With COVID-19

It was suggested that KTRs with moderate COVID-19 infection should withhold antimetabolites and decrease calcineurin inhibitors with or without glucocorticoids [27]. In the setting of severe disease, all maintenance immunosuppression should be stopped, and patients should be started with remdesivir in addition to steroids [27]. This finding was supported by another study with a 30-patient cohort that showed that withholding calcineurin and antimetabolites did not have an increased risk of allograft rejection [28]. Overall treatments included hydroxychloroquine, azithromycin, prednisone, convalescent plasma, and remdesivir [27-33]. For immunosuppression, calcineurin inhibitors, prednisone, and azathioprine were maintained or decreased, while mycophenolate mofetil (MMF) was discontinued, and tacrolimus dosage was reduced during treatment [27-34]. Most patients, despite risk factors for poor outcomes, gradually improved [27-34]. These findings are consistent with previous studies indicating high mortality rates among KTRs. However, in comparison to those studies, the rates of mortality in KTRs with COVID-19 are significantly higher.

In this review, several studies indicated that different treatment regimens affected KTR outcomes, but the overarching theme is that taking patients off immunosuppressants affected them negatively [27-34]. Of 132 COVID-19 patients who were hospitalized and monitored, the overall mortality was found to be 20.5%, with a significant increase (37.8%) in patients needing hospitalization. Of the hospitalized patients, 23% needed kidney replacement therapy and 6.3% lost their transplants [2]. Also, in a study with 30 patients who received a five-day course of hydroxychloroquine and azithromycin, half were tested with methylprednisolone, while the others did not [28]. All patients had discontinued antimetabolites and calcineurin inhibitors. The withdrawal of immunosuppressants in this study showed a mortality rate of 20% without increasing the rate of AKI [28]. Similar results were found when patients were given different treatments that ranged from hydroxychloroquine, azithromycin, convalescent plasma, and remdesivir in a cohort study [29]. As a result, the study had three deaths, and two patients remained hospitalized. Importantly, the mortality rate of COVID-19 KTRs remained high despite the lack of viral coinfections [30]. However, according to Shaikh et al., eight patients developed acute renal allograft dysfunction due to sepsis caused by the immunosuppressed status and related comorbidities [31].

An interesting finding was on African American renal transplant recipients at the Detroit Medical Center experience, which featured a retrospective study of 25 hospitalized patients. They were on tacrolimus and low-dose prednisone but were discontinued from MMF. They were given hydroxychloroquine and steroids, prophylactic anticoagulants, and oxygen supplementation. During follow-up, treatment with mycophenolate was reintroduced. Only one of them required intubation and one died, but overall, they had a mortality rate of 4% compared to the statewide average of 10% [27]. The exact reasons are being explored, but this combination of drugs could lead to better outcomes.

Domain 4: Risk Factors for Increased Mortality in KTRs With COVID-19

Although studies identifying the risk factors for increased mortality rates in KTRs with COVID-19 disease in the United States remain limited, several possible contributing factors have been identified at this time. Immunosuppressive drug regimens, which are part of the standard of care for KTRs, can be associated with an increased risk of severe COVID-19 infection [35]. One small cohort study identified four KTRs who required hospitalization due to COVID-19 infection. Of these four, one patient developed respiratory distress [35]. In another retrospective cohort study, approximately 52% of hospitalized KTRs with COVID-19 infection experienced AKI in addition to respiratory failure as a complication of infection; 29% of these patients required intubation, and the overall mortality rate was found to be 32% [34]. Zimmerman et al. found that 21% of patients requiring hospital admission experienced graft dysfunction and nearly 4% experienced graft failure [36]. This same study found an overall mortality rate of 16.8% in this population [36].

Table 1 reports the characteristics of the 15 primary studies included in this review. Each study is categorized by the following domains: (1) the impact of COVID-19 on performing kidney transplants, (2) the impact of COVID-19 vaccinations on KTRs, (3) the outcomes of various treatment regimens for KTRs with COVID-19, and (4) the risk factors for increased mortality rates in KTRs with COVID-19 in the United States.

**Table 1:** Final summary table of articles in the review. AKI, acute kidney injury; KTR, kidney transplant recipient; UNOS, United Network for Organ Sharing; ARDS, acute respiratory distress syndrome; PCR, polymerase chain reaction; MMF, mycophenolate mofetil

Reference	Purpose	Sample	Study type and description	Major outcomes and limitations
Domain 1: Impact of COVID-19 on performing kidney transplants				
Craig-Schapiro et al. (2021) [23]	Weighing the risks and benefits of transplantation in the setting of ongoing COVID-19	Fifty-six waitlisted patients and 80 KTRs diagnosed with COVID-19 between March 13 and May 20, 2020	Retrospective single-center study	Despite similar demographics and the burden of comorbidities between waitlisted and transplant patients, waitlisted patients were more likely to require hospitalization and were at a higher risk of mortality. Multivariate analysis demonstrated waitlist status, age, and male sex were independently associated with mortality. Intubation portended a very poor prognosis in both groups. Limitations include single-center studies and heterogeneous approaches to patient care due to the limited availability of resources and testing. Not all patients had COVID-19-associated laboratory values checked on admission, and data were incomplete on patients admitted and managed at other hospitals
Chandorkar et al. (2020) [24]	Evaluate the safety of deceased donor kidney transplantation during the covid-19 pandemic	All adult patients who underwent transplantation from March 1, 2020, to April 30, 2020, were included and followed until May 31, 2020.	A single-center retrospective study conducted at Jackson Memorial - Miami Transplant Institute	Seventy-six patients received allografts from 57 donors from March 1, 2020, to April 30, 2020. None of the recipients developed COVID-19 during the period they were followed. A total of 55 readmissions to the hospital. Limitations include possibly missed clinical data such as symptoms of COVID-19 infection due to retrospective study. Relatively small sample size and follow-up period. All COVID-19 testing reported was only done at that center, excluding possible positive results acquired at other facilities.
Domain 2: Impact of COVID-19 vaccinations on KTRs				
Crane et al. (2021) [25]	To evaluate the efficacy of SARS-CoV-2 vaccinations in the KTR population	Twenty-five adolescent kidney transplant patients, with a median age of 19 years and a median time from kidney transplant of five years	Single-center observational study: KTR patients received two doses of an mRNA SARS-CoV-2 vaccine and were tested for the presence of SARS-CoV-2 spike (S) protein antibody presence four to eight weeks after the second dose. KTRs were treated with an immunosuppression regimen, including a calcineurin inhibitor, corticosteroid, and antimetabolite (nine with mycophenolate, three with azathioprine, and one without an antimetabolite due to viremia).	Fifty-two percent of patients had a positive spike antibody. Of those who had an antibody response, fewer had a mycophenolate-containing immunosuppressant regimen than nonresponders. There was a trend toward better vaccine response and higher anti-S antibody titers at lower doses of mycophenolate. These data demonstrate vaccination is safe and supports immunizing KTR who remain hesitant. Future studies should focus on a better understanding of the cellular immune response to vaccination and strategies to enhance vaccine immunogenicity in pediatric solid organ transplant patients.
Husain et al. (2021) [26]	To evaluate postvaccine anti-SARS-CoV-2 spike protein antibody development in KTRs	A total of 28 adult kidney transplant patients with a median age of 66 years and a median time since transplant of 8.7 years	Single-center observational study: Most patients were on tacrolimus, while others were on mycophenolate or belatacept.	Only one-fourth of KTRs who received the vaccine series in full showed a detectable anti-spike protein IgG Ab. However, only two of 17 patients (12%) using mycophenolate at the time of vaccination were antibody-positive, including one with a previous COVID-19 infection and one taking low-dose mycophenolate mofetil (250 mg twice daily). Notably, none of the six patients receiving belatacept were antibody-positive. This response shows KTRs do not show the same immunogenic response that was seen in clinical trials. Limitations include small sample size; cannot conclude vaccine efficacy; IgM, IgA, and T-cell responses were not assessed; patients were not screened for antispike protein antibodies before vaccination.
Domain 3: Outcomes of various treatment regimens for KTRs with COVID-19				
Cruz et al. (2020) [27]	To evaluate outcomes of COVID-19 in African American KTRs in Detroit Medical Center	25 African American renal transplant recipients	Retrospective study Regarding March 1, 2020, to May 1, 2020, COVID-19-positive renal recipient patients. Tacrolimus and low-dose prednisone, while stopping mycophenolate mofetil. Then given hydroxychloroquine and steroids, prophylactic anticoagulants, and oxygen supplementation. At follow-up, treatment with mycophenolate was reintroduced.	All 25 patients were hospitalized. Most of them had comorbidities such as hypertension and diabetes. Only one of them required intubation, and one died but overall, they had a mortality rate of 4% compared to the statewide average of 10%. The exact reasons are being *explored*. One limitation is it is a small sample size, a larger sample size can provide a better understanding.
Chen et al. (2020) [28]	To evaluate immunosuppression management in KTRs with COVID-19 pneumonia	Thirty renal transplant patients with confirmed COVID-19 and admitted to the hospital	Small cohort study: The 30 patients who were admitted received a five-day course of hydroxychloroquine and azithromycin. The patients were then split into two groups - one group received methyl prednisone, while the other did not. Calcineurin inhibitors and antimetabolites were stopped during a hospital stay.	Seven out of the 30 patients in this cohort had to be intubated and were not extubated successfully. However, the other 23 were stable enough to be discharged home. Withdrawal of immunosuppressants in this study showed a mortality rate of 20% without increasing the rate of AKI. Limitations include a small cohort of only 30 patients. The study had a short duration of follow-up, which means a conclusion on the long-term effects of COVID-19 on renal functions cannot be made.
Katz-Greenberg et al. (2020) [29]	To observe how different treatments would affect KTR who tested positive for COVID-19	Twenty KTRs diagnosed with COVID-19 and treated either via hospitalization or outpatient	Single-center observational study: Reduction of immunosuppressants was achieved, and patients were given different treatments that ranged from hydroxychloroquine, azithromycin, convalescent plasma, and remdesivir.	Of the 20 patients in this study, 15 patients were hospitalized. During their stay, a reduction of immunosuppressants was achieved in 50% of the patients. Eleven patients received hydroxychloroquine and four received azithromycin. Four patients received convalescent plasma and three of the four patients were discharged home. There was one patient who received remdesivir as part of their treatment. The study had three deaths, two patients remained hospitalized, and 15 patients were discharged home or managed outpatient. Limitations include a small cohort of patients from a tertiary academic center in Pennsylvania that was not overwhelmed by the surge.
Nair et al. (2020) [30]	To discuss the characteristics, treatment, and evolution of COVID-19 in kidney transplant recipients.	Ten KTRs aged 30-75 years.	Cohort study	Immunosuppression did not reduce the incidence of ARDS or death in COVID-19 kidney transplant patients. The mortality rate of COVID-19 patients remained high, and there were no viral coinfections. This is a small cohort study, and larger studies are needed to fully understand the mortality risk of transplant recipients with COVID-19.
Shaikh et al. (2021) [31]	To explore the occurrence of severe COVID-19 infection in renal transplant recipients admitted to the intensive care unit, their risk factors, severity, course of the disease, and clinical outcomes	Eight postrenal transplant patients with severe COVID-19 infection	Cohort study	Postrenal transplant patients have an increased risk of complications with COVID-19 infection due to their immunosuppressed status and related comorbidities. The eight patients developed acute renal allograft dysfunction due to sepsis. None of the patients had renal transplant rejections, and only three of them received renal replacement therapy in the form of hemodialysis. The antiproliferative agents were discontinued, the calcineurin inhibitors were stopped or inhibited and a higher dose of steroids was administered in all eight patients.
Daniel et al. (2021) [32]	To evaluate outcomes of KTRs after COVID-19	Eighteen kidney transplant patients infected with COVID-19 that underwent allograft surgery	Retrospective study: *For cause* biopsies were done between March 2020 and May 2020. These biopsies were retrospectively chosen only if the patient had a positive nasal swab within 60 days of biopsy or more than 60 days before biopsy without a negative PCR before the biopsy was taken. The samples were placed into two groups.	The most common cause of graft dysfunction in this study is acute rejection. Most biopsies taken during the COVID-19 infection showed increased intraglomerular monocytes, and new development of arteritis when they were compared to biopsies performed before the infection. At the end of the study, only two of the patients in this study demonstrated improved kidney function.
Tejada et al. (2021) [33]	To evaluate outcomes of COVID-19 in African American KTRs in Detroit	Twenty-five African American COVID-positive transplant patients	Case series: Treatments ranged from hydroxychloroquine, azithromycin, convalescent plasma, and remdesivir. For immunosuppression, calcineurin inhibitors, prednisone, and azathioprine were maintained, while MMF was discontinued, and tacrolimus dosage was reduced during treatment.	Twenty-five African American renal transplant patients were diagnosed with SARS from March 1, 2020, to May 1, 2020. They shared many of the common symptoms such as dyspnea and opacities on chest X-rays and were treated similarly with tacrolimus, prednisone, and calcineurin inhibitors. Most of them had comorbidities such as hypertension and diabetes. Most patients, despite risk factors for poor outcomes, gradually improved and made full recovery. The effect of calcineurin on African Americans needs to be further explored.
Azzi et al. (2020) [2]	Investigate the prevalence and clinical outcomes of COVID-19 in KTRs in the Bronx, NY	A total of 1,475 adult kidney transplant recipients	Retrospective cohort study: 1,475 adult KTRs followed at Montefiore Transplant Center, the Bronx, NY. Of this group, 132 patients tested positive for COVID-19 with RT-PCR	Of the 79 patients hospitalized at Montefiore Health System, antimetabolite withdrawal was done in 74 patients (93.7%). Calcineurin inhibitor withdrawal was done in 11 patients (13.9%), mainly following clinical deterioration. Sixty-five patients (82.3%) were treated with antibiotics to prevent secondary infections. All patients were initially started on hydroxychloroquine, but this was discontinued after the first 59 patients due to a lack of efficacy. Forty-four percent of patients received high-dose corticosteroids, 14% received tocilizumab, 7.6% received leronlimab, 13.7% received convalescent plasma, and 55.7% received anticoagulation with apixaban and/or heparin for treatment or prevention. Of the 132 patients who tested positive for SARS-CoV-2 by RT-PCR, 111 patients were hospitalized (79 at Montefiore Medical Center and 32 at outside facilities), and 21 were monitored as outpatients. Overall mortality was found to be 20.5%, with a significant increase (37.8%) in patients needing hospitalization. Of the hospitalized patients, 23% needed kidney replacement therapy and 6.3% lost their transplants. The study did not assess the longevity of the IgG antibodies in the patient population and only provided qualitative IgG testing (quantitative testing would have provided additional information). Additionally, they did not test for IgM antibodies to SARS-CoV-2. Risk factors for increased mortality rates with COVID-19 included older age, receipt of a deceased donor kidney transplant, renal disease due to diabetic nephropathy, diabetes mellitus, no immunization for influenza the previous year, and higher levels of serum IL-6.
Domain 4: Risk factors for increased mortality rates in kidney transplant patients with COVID-19				
Cravedi et al. (2020) [34]	To identify the outcomes of a large multicenter cohort of 144 kidney recipients who were hospitalized due to COVID-19 across 12 transplant centers in North America and Europe to point out the predictors of poor clinical outcomes	9,845 adult kidney transplant patients, of which 144 were hospitalized for COVID-19 in a nine-week study	Retrospective cohort study: Therapeutic management: antimetabolite withdrawal (68%), calcineurin inhibitor withdrawal (23%), hydroxychloroquine (71%), antibiotics (74%), tocilizumab (13%), and antivirals (14%)	During a median follow-up after 52 days, AKI occurred in 52% of cases in addition to respiratory failure that warranted intubation in 29%. The mortality rate was 32%. Of this large cohort that was tracked after their hospitalization, they had a higher rate of AKI and mortality, with new targets to investigate like optimal treatment and the role of the common inflammatory markers that were noted. However, the selection criteria limit the generalizability of these findings as they are only targeting infected hospitalized patients, so conclusions about all kidney transplant patients cannot be made.
Santeusanio et al. (2020) [35]	To assess the safety and outcomes of proceeding with new kidney transplants and current standards of care in the setting of the COVID-19 pandemic	Thirty adults who received kidney transplants at a single center in New York City during the height of the COVID-19 pandemic in 2020	Observational study of care and outcomes of 30 adult patients who received a kidney transplant during the COVID-19 pandemic at a single center in New York, NY	Kidney transplantation in areas endemic to COVID-19 using standard induction and maintenance immunosuppression appears to be associated with a modest risk for severe COVID-19-related disease. Of the 30, four following patients were readmitted posttransplant for COVID-19 infection. A disease in one of these patients led to respiratory distress.
Zimmerman et al. (2020) [36]	To identify potential differences in the impact of COVID-19 on kidney transplant patients in regions other than epicenters of the pandemic	A total of 6,568 adult kidney transplant patients from 13 centers in the UNOS region 1	The survey-based study identified 189 COVID-19 cases and outcomes among 6,568 kidney transplant patients across 13 centers.	This study estimated an incidence of COVID-19 in kidney transplant patients of 2.88%. Of the patients who tested positive for COVID-19 infection, 137 required hospital admission, 29 experienced graft dysfunction, 5 experienced graft failure, and 23 died.

Discussion

Performing Kidney Transplants on Patients With COVID-19

According to the literature reviewed, undergoing kidney transplantation might provide a protective effect if the recipient was infected with COVID-19 when compared to those infected who were waiting for a kidney [23,24]. This is evident from the increased rates of hospital admissions of waitlisted COVID-19-positive patients, compared to hospitalizations of COVID-19-positive KTRs [23]. The basis of this observation could be due to the transplantation procedure itself posing an elevated COVID-19 infection risk, and a new functioning kidney being associated with improved infection outcome [23,24]. Further, the improved outcome of KTRs compared to those waitlisted could also be attributed to the immunosuppressive regimen hindering the development of severe symptoms secondary to the viral infection [24]. There have been some preliminary studies suggesting the benefits of certain therapies to decrease the risk of cytokine storm in patients with COVID-19 who have acute respiratory distress syndrome (ARDS) [23].

COVID-19 Vaccinations and KTRs

Regarding the impact of COVID-19 vaccinations on KTRs, the overall effect of vaccination in terms of the immune response produced by the vaccine seems to depend on which immunosuppression regiment the patient was following. Adolescent patients receiving either lower doses of mycophenolate or an immunosuppressant regimen that did not contain mycophenolate, recorded a stronger immune response compared to patients who were nonresponsive to the vaccination who received higher doses of mycophenolate [25].

COVID-19 vaccination appears to be safe for KTRs, but immunoglobulin levels can vary depending on the immunosuppressive regimen. Given this, if feasible, it might be an effective approach to temporarily switch a patient’s immunosuppressive regimen before vaccination to maximize the immune response. A valid pharmacological regimen could include low-dose mycophenolate as it is associated with higher spikes in immunoglobulin levels. The use of belatacept as part of immunosuppressive therapy should be carefully evaluated, given the negative association with antispike protein antibody levels. This is important to consider for future vaccines targeting new COVID-19 variants. These studies have shown that the immune response KTRs have from the COVID-19 vaccine differs from the general population and vary depending on which immunosuppressive regimen the patient is receiving [25,26].

Treatment Regimens for KTRs With COVID-19

Several studies reported that a reduction of immunosuppressive therapy increased mortality rates of hospitalized patients, except in the specific situation of African American KTRs [33]. This finding emphasizes the need to develop individual-oriented management strategies in treating KTRs with COVID-19. Further, many of these treatments included the reduction in mycophenolate/tacrolimus with the addition of azithromycin and hydroxychloroquine [32,33]. Interestingly, there was an increased rate of mortality in patients who were withdrawn from immunosuppressive therapy. This could be consistent with an immune response affecting the patient beyond their capacity, leading to death, although they did not develop AKI. The concept of removing patients from immunosuppression altogether does not seem to prove effective, and a reduction in pharmacology might be a better approach. More studies need to be done about the specific factors that affected the increased and decreased mortality rates to rule out immunosuppression withdrawal as an ineffective management strategy. Further, many of the studies analyzed contain overlapping medications; however, each test group might not have been entirely comparable, given that they were on other medications as well. Given the uncertainty in the interactions between all the drugs that the patients were on, it would be premature to conclude that the patient is on any specific drug that had a protective effect.

Risk Factors for Increased Mortality in KTRs With COVID-19 

Two studies in this review indicated that certain risk factors were found to be more strongly associated with KTR mortality rates [32,35]. As expected, markers of AKI showed a significant predictive value of transplant failure and mortality. Lower glomerular filtration rate, high serum lactate dehydrogenase, C-reactive protein (CRP), creatinine, D-dimer, procalcitonin, and IL-6 have been some the markers associated with mortality in hospitalized KTRs with COVID-19 [32]. These risk factors underline the need for KTRs to obtain hospital protocols to follow in the case of an infection, to avoid a late presentation with exacerbated symptoms. Interestingly, concomitant HIV infection has also been determined to be associated with increased mortality, while immunosuppression pharmacology and adjustment following COVID-19 infection are, independently, not correlated with acute rejection or mortality [35]. Finally, age alone was a significant risk factor in surviving patients, indicating the need to focus on prevention, management, and education of the KTR population aged over 60 years [34]. However, of the studies that are currently available, many agree that immunosuppressive drug therapy administered to KTRs may be a potential contributing factor impacting the mortality in this population.

AKI markers, although highly correlated with mortality, might not be modifiable targets in the assessment of the KTRs with COVID-19. However, the time of hospital presentation with lymphopenia and hypoxemia could be a target in protocol development. Educational material for KTRs should target awareness of the time dependence of their condition and the need for early intervention/presentation to decrease mortality risk. Further, the type of immunosuppressive pharmacology was not associated with higher mortality or rejection; therefore, attempts to switch patients from medical regimens might not prove an effective strategy in minimizing disease impact. Instead, other methods such as the addition of medications that target the virus or the reduction of an immunosuppressive regimen might be more promising in managing COVID-19-infected KTRs.

Limitations of the Included Studies

First, only articles published in English were selected due to the limited availability of translation services. Second, the timeframe of the inclusion criteria limited the selection to articles published on or after 2020 to 2021; therefore, articles published in late 2019 when mentions of COVID-19 first started were not included. The inclusion criteria also limited the selection to articles from primary sources only. As COVID-19 is a relatively new disease, the current research regarding potential risk factors impacting mortality rates among KTRs with COVID-19 infection is still limited. Many currently published studies focus on small populations across specific geographic locations, resulting in limited information on the presentation and poor outcomes of the population in question and reduced access to resources necessary to identify potential differences influencing COVID-19 infection. Of note, the studies utilized in this scoping review did not appear to contain any strong and direct contradictions across their findings.

Limitations of the Review Process

Limitations of this review process include using a finite number of databases and focusing specifically on the COVID-19 infection in KTRs in the United States. Also, articles were selected based on primary data only and those that were published in or after 2019. Other limitations include time and resources to cover all the available articles.

Implications for Future Research

A valid future endeavor would be to further explore the correlation between hypoxemia at the time of presentation and mortality levels. Respiratory failure was also discussed as a relevant complication along with organ failure; therefore, it would be an interesting approach to assess how early oxygenation in infected patients, whether admitted to a hospital or not, could change the disease course. Further, lymphopenia was also an associated risk factor for mortality, which can be explored. It would be important to know whether these patients were in a prior pharmacologically induced lymphopenia state and whether vaccination status would have a protective or delayed effect in the development of lymphopenia after COVID-19 infection. Given the increase in mortality rate in patients with HIV, more research is needed to explore the disease course in patients with immunosuppressive comorbid conditions and their response to the current treatment protocol of temporarily decreasing immunosuppressive therapy for COVID-19 recovery. In short, more research is needed to analyze how these mortality-correlated factors can be modified to minimize disease impact. To acquire a more thorough overall understanding of the immune response seen in KTRs from the COVID-19 vaccines, large studies evaluating IgM, IgA, and T-cell responses in KTRs should be done.

## Conclusions

Analysis of the 16 identified publications in this review demonstrated kidney transplantation, and the procedure’s accompanying immunosuppressant regimen provided KTRs with better COVID-19 infection outcomes when compared to waitlisted patients. Hospitalization, graft dysfunction, AKI, and respiratory failure were the most common risk factors that increased the risk of mortality in COVID-19-positive KTRs. This review also illustrated withdrawing KTRs from immunosuppressive drugs was not effective in improving outcomes in patients with active COVID-19 infection. Further studies are needed to research the effect of specific drugs and dosages on the severity and mortality rate of COVID-19 in KTRs and will provide more robust suggestions for clinical practice in minimizing the impact of COVID-19 infection.
